# Undernutrition among HIV-positive children in Dar es Salaam, Tanzania: antiretroviral therapy alone is not enough

**DOI:** 10.1186/1471-2458-11-869

**Published:** 2011-11-16

**Authors:** Bruno F Sunguya, Krishna C Poudel, Keiko Otsuka, Junko Yasuoka, Linda B Mlunde, David P Urassa, Namala P Mkopi, Masamine Jimba

**Affiliations:** 1 Department of Community and Global Health, Graduate School of Medicine, The University of Tokyo, 7-3-1, Hongo, Bunkyo-ku, Tokyo, 113-0033, Japan; 2 School of Public Health and Social Sciences, Muhimbili University of Health and Allied Sciences, P.O Box 65489, Dar es Salaam, Tanzania; 3 Department of Pediatric and Child Health, Muhimbili National Hospital, P.O Box 65000, Dar es Salaam, Tanzania

## Abstract

**Background:**

The prevalence of HIV/AIDS has exacerbated the impact of childhood undernutrition in many developing countries, including Tanzania. Even with the provision of antiretroviral therapy, undernutrition among HIV-positive children remains a serious problem. Most studies to examine risk factors for undernutrition have been limited to the general population and ART-naive HIV-positive children, making it difficult to generalize findings to ART-treated HIV-positive children. The objectives of this study were thus to compare the proportions of undernutrition among ART-treated HIV-positive and HIV-negative children and to examine factors associated with undernutrition among ART-treated HIV-positive children in Dar es Salaam, Tanzania.

**Methods:**

From September to October 2010, we conducted a cross-sectional survey among 213 ART-treated HIV-positive and 202 HIV-negative children in Dar es Salaam, Tanzania. We measured the children's anthropometrics, socio-demographic factors, food security, dietary habits, diarrhea episodes, economic status, and HIV clinical stage. Data were analyzed using both univariate and multivariate methods.

**Results:**

ART-treated HIV-positive children had higher rates of undernutrition than their HIV-negative counterparts. Among the ART-treated HIV-positive children, 78 (36.6%) were stunted, 47 (22.1%) were underweight, and 29 (13.6%) were wasted. Households of ART-treated HIV-positive children exhibited lower economic status, lower levels of education, and higher percentages of unmarried caregivers with higher unemployment rates. Food insecurity was prevalent in over half of ART-treated HIV-positive children's households. Furthermore, ART-treated HIV-positive children were more likely to be orphaned, to be fed less frequently, and to have lower body weight at birth compared to HIV-negative children.

In the multivariate analysis, child's HIV-positive status was associated with being underweight (AOR = 4.61, 95% CI 1.38-15.36 *P = 0.013*) and wasting (AOR = 9.62, 95% CI 1.72-54.02, *P = 0.010*) but not with stunting (AOR = 0.68, 95% CI 0.26-1.77, *P = 0.428*). Important factors associated with underweight status among ART-treated HIV-positive children included hunger (AOR = 9.90, *P = 0.022*), feeding frequency (AOR = 0.02, *p < 0.001*), and low birth weight (AOR = 5.13, *P = 0.039*). Factors associated with wasting among ART-treated HIV-positive children were diarrhea (AOR = 22.49, *P = 0.001*) and feeding frequency (AOR = 0.03, *p < 0.001*).

**Conclusion:**

HIV/AIDS is associated with an increased burden of child underweight status and wasting, even among ART-treated children, in Dar es Salaam, Tanzania. In addition to increasing coverage of ART among HIV-positive children, interventions to ameliorate poor nutrition status may be necessary in this and similar settings. Such interventions should aim at promoting adequate feeding patterns, as well as preventing and treating diarrhea.

## Background

Undernutrition is an underlying factor in around 35% of preventable deaths among under-five children [1]. In Sub-Saharan Africa, the magnitude of nutritional problems varies across the region, with proportions of stunting and wasting as high as 32% and 10%, respectively [2]. Such high undernutrition rates have resulted from multiple hardships including food-insecurity, poverty, and rampant diseases, especially HIV/AIDS [3]. HIV/AIDS indeed has increased the severity of pre-existing undernutrition cases. Consequently, over half of children with HIV/AIDS may also be suffering from severe undernutrition [4]. Studies that compared nutrition status in Sub-Saharan Africa have shown a higher proportions of underweight status [5,6], wasting [6,7], and stunting [5-7] among HIV-positive compared to among HIV-negative children.

Both HIV/AIDS and undernutrition affect immune function, with lack of essential micronutrients leading to nutritionally acquired immune-dysfunction syndrome [8,9]. Compromised immune defenses increase susceptibility to infectious diseases and complicate case management [10]. Under such conditions, case fatality rates in children are prone to increase even under the standard treatment guidelines of the World Health Organization (WHO) [11-13]. Initiation of antiretroviral therapy (ART) improves immunity, enables the body to fight opportunistic infections, and reduces energy loss. In this way, nutritional deficiencies can be ameliorated [14]. ART alone, however, may not be sufficient to revive an already compromised nutritional status. Despite current global efforts to increase ART coverage, HIV-infected children remain nutritionally challenged due to socio-economic, disease, and other specific health-related factors [15].

The socio-economic risk factors for undernutrition are known to be associated in children with ART-naïve HIV-positive status. Such factors include low economic status and orphanhood [16], food insecurity [17], poor dietary patterns [18], and low maternal education [17]. Additionally, diarrhea is also implicated as a risk factor for undernutrition among ART-naïve HIV-positive children [16]. Health-related factors associated with undernutrition among ART-naïve HIV-positive children include low CD_4 _count and high viral load [8,19]. In addition to recognized risk factors for undernutrition, most studies have been conducted among HIV-positive children who are not yet under ART. Thus, it has been difficult to generalize findings to ART-treated HIV-positive children.

Tanzania is one of a number of countries devastated by dual burdens of HIV/AIDS [20] and undernutrition among children [21]. Despite the increased number of children enrolled in HIV/AIDS care and treatment facilities [20], HIV-positive children continue to suffer from an unacceptably high undernutrition toll [21]. The national HIV/AIDS prevalence is 5.7% [20]. Meanwhile, in the general population, 47.8% of children are stunted, while 7.0% and 4.5% of children suffer from underweight and wasting, respectively [22]. In a study conducted in Dar es Salaam in 2004, a time when ARV was not nationally available, HIV-positive children had a higher proportion of stunting [7]. However, we found no evidence of similar studies conducted elsewhere among ART-treated HIV-positive children. Furthermore, studies on determinants of undernutrition are limited to only one study in Tanzania [23]. In this study, children of HIV-positive mothers were included without taking their HIV status into account. Moreover, evidence on nutritional status among ART-treated HIV-positive children in Tanzania is also lacking. Therefore, the objectives of this study were (i) to compare the rates of undernutrition among ART treated HIV-positive and HIV-negative children, and (ii) to examine factors associated with undernutrition among ART-treated HIV-positive children in Dar es Salaam, Tanzania.

## Methods

### Study design and area

This cross-sectional study was conducted among HIV-negative and ART-treated HIV-positive children in Dar es Salaam, Tanzania. Nutritional status among ART-treated HIV-positive children was compared with that of HIV-negative children. In 2010, Dar es Salaam reported that about 18.8% of children in the general population were stunted, 11.8% were underweight, and 6.8% were wasted [22].The HIV prevalence in the city was 8.9% in 2009, which was higher than the national average [20]. While approximately 7,000 HIV-positive children in Dar es Salaam were attending Care and Treatment Centers (CTCs); only about 4,000 children were under ART [20]. Dar es Salaam has 44 health facilities with ART programs, the majority of which are run by the government and collaborating partners [24].

According to the national guidelines for HIV care and treatment in Tanzania, which were in use during this survey, ART treatment initiation depends on disease progression [24]. Since disease progression differs among children, the guidelines also demand individualization at initiation of treatment based on biological and social factors. Pertinent biological factors include age, HIV-related diseases, immunosupression, and viral load. Social factors include disclosure status, caregiver's commitment, and family support. All HIV-positive children under 12 months of age are supposed to start ARV, while for HIV-positive children older than 12 months, both clinical and immunological threshold were used as indicators. Such criteria include WHO clinical stages 1 and 2 with CD4 <25% or WHO clinical stage 3 or 4, irrespective of CD4%. [24].

ART-treated, HIV-positive children in Tanzania are typically treated by combination therapy incorporating three ARVs. The recommended fixed combination of ART is either two Nucleoside Reverse Transcriptase Inhibitors (NRTI) and a Non-Nucleoside Reverse Transcriptase Inhibitors (NNRTI) or two NRTI and Boosted Protease inhibitor (PIr). The appropriate combination also varies with age, where, for children younger than 36 months of age, a combination therapy includes Zidovudine, Lamivudine and Niverapine. Children older than 36 months receive a combination of Zidovudine, Lamivudine and Efavirenz. In the event of Nevirapine exposure such as during pregnancy for PMTCT, Nevirapine is usually substituted with Lopinavir boosted with Ritonavir. When a child presents with anemia, Zidovudine is exchanged for Stavudine. A monthly visit for ARV refills ensures adherence, monitoring of side effects, and early detection of treatment failure [24].

### Participants

We recruited 435 pairs of children aged 6-60 months, along with their caregivers. For the HIV-positive group, only those under ART were selected. ART-treated, HIV-positive children were selected from HIV care and treatment centers (CTCs) where they undergo treatment. Upon their enrollment or referral to CTCs, several diagnostic tests are conducted to confirm their sero-status. For children under 18 months of age, DNA-PCR test is done to avoid false-positive results due to persistent maternal antibodies. A PCR machine is housed within the specialized laboratory of Muhimbili National Hospital. The collected Dried Blood Samples (DBS) are sent to this laboratory for diagnosis. The algorithm for HIV/AIDS diagnosis among children older than 18 months is through three rapid antibody tests which are Determine^® ^(Abbot, Wiesbaden, Germany), SD Bioline HIV-1/2^® ^(Standard Diagnostics, Kyonggi-do, South Korea) and Uni-gold^® ^(Trinity Biotech, Bray, Ireland) in the CTC. Enzyme-linked Immunosorbent Assay (ELISA) may also be used under special circumstances where rapid antibody tests could not facilitate definitive diagnosis. However, a specialized laboratory and technicians are required for such diagnostic [24]. We excluded children with missing ART information and those whose caregivers did not consent to participate.

For the comparison group, we recruited HIV-negative children and their caregivers from general pediatric clinics in Dar es Salaam. General pediatric clinics are part of Reproductive and Child Health (RCH) clinics, which under-five children with or without ailments attend for growth monitoring and vaccination. Children who presented at the clinic with minor ailments, such as fever, diarrhea, or upper respiratory infections, were excluded from this study. To group these children as an HIV-negative group, we relied on self-reported information from the caregiver, medical files and child follow-up cards, child's past medical history, reported HIV/AIDS results from mother during antenatal care, and physical examination of the child. From this group, we excluded children with known HIV-positive status and those whose parents were known to have HIV/AIDS or to have died of HIV/AIDS-related illnesses.

HIV-positive participants were recruited from health facilities providing free ART services to over 100 children. In total, 12 out of a total of 44 facilities with ART programmes in the region fit the selection criteria. Three health centers were dropped due to their peripheral location. Muhimbili was also dropped because it is the city's referral hospital. Five centers that provide nutrition supplements to children with severe undernutrition were also excluded. The final three health facilities selected for the study were thus Buguruni, AKC, and Sinza CTCs. To recruit children for the control group, we selected reproductive and child health clinics from a list of health facilities with ART programmes by convenience sampling. In this study, we selected AKC and Sinza RCH clinics for recruitment of HIV-negative children. The selected health facilities consisted of both CTCs and RCHs serving the three Dar es Salaam municipalities - Ilala, Kinondoni, and Temeke. With respect to access, a client from any municipality may attend any of the facilities inside the municipality or other areas.

We selected HIV-positive participants from each respective CTC health facility by systematic random sampling using a prepared register of eligible children. We chose one participant after every other eligible one in the prepared roster for participation in the study. When the participant refused to participate after being chosen, we proceeded on to the next individual on the list. For the HIV-negative group, we used random sampling based on daily attendance, with a minimum of 15 children recruited for one day. The recruited participants from both CTCs and RCHs represented the three municipalities, i.e. Ilala (HIV-positive-79, HIV-negative-70), Kinondoni (HIV-positive-101, HIV-negative-106) and Temeke (HIV-positive-33, HIV-negative-26). About the participants per clinic, we recruited: 84 HIV-positive participants from AKC CTC and 99 HIV-negative participants from its RCH clinic; 76 HIV-positive participants from Sinza CTC and 103 HIV-negative participants from its RCH clinic; and 53 HIV-positive participants were recruited from Buguruni CTC. All the selected facilities are run by the government and have similar standard operating procedures.

We used Power and Precision version 4 (Biostat, Englewood, NJ, USA) software to calculate the minimum sample size that would detect the difference in the proportion of undernutrition between HIV-positive and HIV-negative children at a power of 80, with 95% CI and a standard deviation of 17. As information on the prevalence of undernutrition among ART treated HIV-positive children in Tanzania or Sub-Saharan Africa was not available, we used data from a study conducted in Dar es Salaam [7] among HIV-positive and HIV-negative children. In this study, the proportion of stunting among HIV-positive and HIV-negative children was 35.6% and 29.6%, respectively. Relevant proportions for underweight and wasting among the two groups could not be found. Therefore, the estimated minimum sample size for each group was calculated to be 183. In this study, we recruited a total of 435 children, among whom 233 were HIV-positive and 202 HIV-negative. The data for 20 ART-treated HIV-positive children were excluded due to missing data regarding ART and errors in anthropometric information.

### Measurements

The children's weights were measured using a standardized hanging Salter scale^® ^(UK) calibrated to 0.1 kg for children who could not stand and a standardized Seka^® ^digital scale (Brooklyn, USA) for children who could stand. We measured height using a Seka^® ^measuring rod calibrated to 0.5 cm. Weight and height were converted to weight-for-age z-score (WAZ), weight-for-height z-score (WHZ) and height-for-age z-score (HAZ) using Epi-Info/ENA version 3.5.1, 2008 (CDC, Atlanta, Georgia, USA) software, according to WHO reference values [25]. Based on the recommendations of the WHO Global Database on Child Growth and Malnutrition, z-scores <-2SD and <-3SD defined moderate and severe undernutrition, respectively.

Socio-demographic variables pertaining to children and their caregivers were adopted from the Tanzania Demographic and Health Survey (TDHS), women and household questionnaires [21,22]. A caregiver was defined as the child's caretaker, parent, or a guardian that accompanied the child to the clinic. Information collected included education level, orphanhood, religion, and marital status. A child was considered an orphan if he/she had lost one or both parents.

Food security was assessed using the short-form HFSS [26], a 6-item scale developed in the United States (US) from the original 18-item scale [27] and used in various settings including the US [28], Bolivia, Burkina Faso, the Philippines [29], and the Caribbean [30]. This scale is used to measure household food security by 12-month recall. Characterization is based on the sum of affirmative responses; two or more affirmatives indicates 'food insecurity', while 5 or more affirmatives indicates 'hunger'. In the present study, Cronbach's alpha for the HFSS was 0.72, with corrected item-total correlation ranging from 0.09 to 0.74.

A total dietary diversity score was calculated from a recalled list of food items consumed over the previous day. Based on a set list of 12 food items, a score lower than 6 was classified as low dietary diversity [31]. Listed food items were adopted from the TDHS, child questionnaire [21,22]. Frequency of feeding was assessed by a 24-hour feeding recall; feeding frequency of less than 4 (median) was considered low.

Economic status was assessed by using a Weighted Wealth Index incorporating household assets ownership, housing characteristics, fuel for lighting and cooking, type of toilet, source of water, and feeding characteristics [21,22]. Dichotomous variables were constructed and factor analysis using principle component analysis (PCA) used to reduce 42 items to 22 (loaded as factor 1). Factor loadings were used as item weights, which were totaled to yield the wealth index for each household [32-34]. The total Weighted Wealth Index score was then equally divided into terciles designating high, middle, and low economic status.

Children registering a WHO clinical HIV stage above two were regarded as advanced cases [35]. In this study, we extracted the highest reached WHO clinical HIV stage from the medical file of each child. Such information is routinely updated on every CTC visit.

### Data collection

Eight nurse counselors were hired and trained for one day on interviewing technique and questionnaire content. They thereafter pre-tested the questionnaire under supervision of the first author. Data were collected by face-to-face interview and relevant medical data were retrieved from medical files in September and October 2010.

### Data analysis

We analyzed data from 213 ART-treated HIV-positive children and 202 HIV-negative children. Descriptive analysis was conducted by Chi-square and independent sample T-tests. Both bivariate and multiple logistic regression analyses were used to examine associations of various factors with wasting, underweight, and stunting. Multiple logistic regression analyses were used to examine the associations between children's HIV status and underweight, wasting, and stunting, respectively. In these models, we controlled for child's age, sex, birth weight, dietary diversity score, ever breastfed status, caregiver's age and education level, employment status, food security, and wealth index score. We also used multiple logistic regression to examine the factors associated with nutritional status (underweight, wasting, and stunting) among ART-treated HIV-positive children alone. Variables included in these multivariate analyses models were as follows: age, sex, birthweight, diarrhea episodes in the past six months, caregiver's age, caregiver's education level, feeding frequency, HIV stage, household food security, household wealth index, dietary diversity score, and ART duration. From the regression models, we excluded 'malaria episodes' and 'hospitalization in the past six months' variables because they showed high correlations with diarrhea (R = 0.5 and R = 0.6, respectively). 'Orphanhood' and 'total months breastfed' variables were also excluded due to missing values. Multicollinearity in both models was checked by examining the standard errors for regression coefficients. Statistical significance was set at *P-value*<0.05. Analysis was conducted using PASW 18 (SPSS Inc., Chicago, Illinois, USA).

### Ethical considerations

This study was approved by the Institutional Review Boards of The University of Tokyo and the Muhimbili University of Health and Allied Sciences, Dar es Salaam. Permission to conduct the research was granted by the relevant municipality health departments and by the managing medical officer in the health facilities. Participation was voluntary, confidentiality ensured, and informed consent secured before the start of each interview.

## Results

### Descriptive results

The mean age of ART-treated HIV-positive children was significantly higher than that of HIV-negative children (Table 1). Compared to HIV-negative children, higher proportions of ART-treated HIV-positive children were orphaned (*p < 0.001*), born with low weight (*P = 0.008*), and presenting with a lower food diversity score (*p < 0.001*). Compared to HIV-negative children's caregivers, a higher proportion of ART-treated HIV-positive children's caregivers had low education attainment (*p < 0.001*), unemployment status (*p < 0.001*), and unmarried status (*p < 0.001*). Compared to HIV-negative children, a higher proportion of ART-treated HIV-positive children were living in households with a low wealth index (*p < 0.001*) and higher proportions of food insecurity and hunger (*p < 0.001*).

**Table 1:** Descriptive analysis: Child, caregiver, household characteristics and undernutrition

	HIV Negative (N = 202)				HIV Positive (N = 213)				
Variable	N	Mean	%	SD	N	Mean	%	SD	P-Value
**Age child**(Months)		14.5		9.6		38.6		15.6	< 0.001
6-12 months	115		56.9		15		7.0		
13-24 months	62		30.7		29		13.6		< 0.001
>24 months	25		12.4		169		79.3		
**Sex**									
Male	109		54.0		113		53.1		0.931
Female	93		46.0		100		46.9		
**Orphan hood**									
Yes	2		1.0		59		27.7		< 0.001
No	200		99.0		154		72.3		
**Birth weight**									
≥2500 g	169		83.7		154		72.3		0.008
<2500 g	33		16.3		59		27.7		
**Age caregiver**(Years)		27.8		5.4		33.0		8.1	< 0.001
**Caregiver's education**									
≤ Primary	110		54.5		165		77.5		< 0.001
≥ Secondary	92		45.5		48		22.5		
**Major religions**									
Christian	93		46.0		102		47.9		0.255
Moslem	109		54.0		111		52.1		
**Employment status**									
Not employed	103		49.0		148		69.5		<0.001
Employed	99		51.0		65		30.5		
**Marital status caregiver**									
Married	182		90.1		164		77.0		<0.001
Not married	20		9.9		49		23.0		
**Wealth index (tercile)**									
High	102		50.5		57		26.8		<0.001
Middle	59		29.2		69		32.4		
Low	41		20.3		87		40.8		
**Food security score (0-6)**									
Secured	150		74.3		101		47.4		< 0.001
Insecure	26		12.9		35		16.4		
Hunger	26		12.9		77		36.2		
**Dietary diversity score**									
≥ 6	132		65.3		95		44.6		<0.001
< 6	70		34.7		118		55.4		
**Currently breastfed**									
Yes	142		70.3		3		1.4		<0.001
No	60		29.7		210		98.6		
**Diarrhea(past 6 months)**									
Yes	35		17.3		82		38.5		<0.001
No	167		82.7		131		61.5		
**Malaria (past 6 months)**									
Yes	46		22.8		83		39.9		0.001
No	156		77.2		130		61.0		
**Hospitalization(past 6 months)**									
Yes	16		7.9		61		28.6		<0.001
No	186		92.1		152		71.4		
**ART Duration (months)**						18.0		12.40	
**WAZ (Underweight)**									
Normal	189		93.5		166		77.9		<0.001
Moderate	9		4.5		33		15.5		
Severe	4		2.0		14		6.6		
**WHZ (Wasting)**									
Normal	200		99.0		184		86.4		<0.001
Moderate	2		1.0		20		9.4		
Severe	0		0.0		9		4.2		
**HAZ (Stunting)**									
Normal	158		78.2		135		63.4		0.004
Moderate	29		14.4		49		23.0		
Severe	15		7.4		29		13.6		

Compared to only 3 (1.4%) HIV-positive children who were still breastfed, 142 (70.2%) children in the HIV-negative group were still being breastfed at the time of data collection. ART-treated, HIV-positive children had higher proportions of diarrhea *(p < 0.001)*, malaria *(P = 0.001)*, and hospitalization *(p < 0.001) *during the six months prior to data collection compared to HIV-negative children.

### Children's nutritional status

The HIV-positive children under ART had higher rates of all forms of undernutrition compared with their HIV-negative counterparts. About 22% and 13%, respectively, of ART-treated HIV-positive children were underweight and showed signs of wasting. Similarly, about 37% of ART-treated HIV-positive, compared to about 22% of HIV-negative children, were stunted. (Table 1)

### Association of HIV/AIDS status and nutrition status

Compared to HIV-negative children, ART-treated HIV-positive children were more likely to have underweight (4.61, 95% CI 1.38-15.36 *P = 0.013*) and to show signs of wasting (AOR = 9.62, 95% CI 1.72-54.02). However, stunting was not associated with HIV-positive status after adjusting for important confounders (AOR = 0.68, 95% CI 0.26-1.77, *P = 0.428*) (Table 2).

**Table 2:** Multiple Logistic Regression, The association of HIV and nutrition status

		WAZ <-2SD				WHZ<-2SD				HAZ<-2SD			
Variables	N	AOR	95% C.I.for AOR		P-Value	AOR	95% C.I.for AOR		P-Value	AOR	95% C.I.for AOR		P-Value
			**Lower**	**Upper**			**Lower**	**Upper**			**Lower**	**Upper**	
**HIV**													
Negative	202	1.00				1.00				1.00			
Positive	213	4.61	1.38	15.36	0.013	9.62	1.72	54.02	0.010	0.68	0.26	1.77	0.428
													

Adjusted for Child's age, sex, Birth weight and Dietary diversity score; Caregiver's age, education level and employment status; Household's wealth index and food security; and Ever breastfeeding.

### Association of ART duration and nutrition status

The mean duration of ART among HIV-positive children was 18.2 months (standard deviation, 12.4 months) (Table 1). Among children on ART for less than 6 months, 13 (27.7%) were underweight, compared to 13 (33.3%) among those on ART for a duration between 6 and 12 months and 21(16.5%) among those on ART for more than 12 months (*P = 0.054*). Wasting was detected in 5 (10.6%) children on ART for less than 6 months compared to 8 (20.5%) and 16 (12.6%) among children on ART for a duration of 6 to 12 months and more than 12 months, respectively (*P = 0.388*). Similarly, 19 (40.4%) children on ART for less than 6 months were stunted, compared to 19 (48.7%) and 40 (31.5%) children on ART for 6 to 12 months and more than 12 months, respectively (*P = 0.127*).

### Determinants of undernutrition among ART-treated HIV-positive children

Table 3 shows the results for factors associated with undernutrition among ART-treated HIV-positive children in the multiple logistic regression analyses. Higher risk of being underweight was observed among children from households under conditions of hunger (AOR = 8.64, 95% CI 1.26-59.23, *P = 0.028*) and among those born with low weight (AOR = 5.13, 95% CI 1.10-24.28, *P = 0.039*), while lower risk of being underweight was associated with higher feeding frequency (AOR = 0.02, 95% CI 0.01-0.11, *p < 0.001*). Households with middle wealth index scores were less likely to have children with underweight status compared to households with low wealth index scores (AOR = 0.08, 95% CI 0.0.01-0.58, *P = 0.013*).

**Table 3:** Multiple Logistic Regression; Risk factors of Undernutrition of HIV positive children.

	WAZ<-2SD					WHZ<-2SD					HAZ<-2SD				
Variable	OR	AOR	95% C.I.for AOR		P-Value	OR	AOR	95% C.I.for AOR		P-Value	OR	AOR	95% C.I.for AOR		P-Value
			Lower	Upper				Lower	Upper				Lower	Upper	
**Age**															
≤38 Months(median)	1.00	1.00				1.00	1.00				1.00	1.00			
>38 Months(median)	1.51	0.48	0.06	3.56	0.469	1.18	0.94	0.19	4.68	0.94	0.90	0.84	0.24	2.95	0.781
**Sex**															
Male	1.00	1.00				1.00	1.00				1.00	1.00			
Female	0.45	0.54	0.13	2.29	0.403	0.46	0.33	0.09	1.17	0.085	0.63	0.90	0.40	2.06	0.810
**Birth weight**															
≥2500 g	1.00	1.00				1.00	1.00				1.00	1.00			
<2500 g	5.65	5.13	1.10	24.28	0.039	2.44	0.63	0.17	2.39	0.495	3.40	1.09	0.42	2.83	0.865
**Diarrhea**															
No	1.00	1.00				1.00	1.00				1.00	1.00			
Yes	35.01	3.12	0.57	17.19	0.191	31.66	22.49	3.38	149.61	0.001	7.75	1.28	0.43	3.82	0.653
**Education**(caregiver)															
≤ Primary	1.00	1.00				1.00	1.00				1.00	1.00			
≥ Secondary	0.26	0.14	0.02	1.25	0.076	0.51	0.90	0.14	5.30	0.919	0.50	0.73	0.22	2.06	0.465
**Feeding frequency**															
<4	1.00	1.00				1.00	1.00				1.00	1.00			
≥4	0.02	0.02	0.01	0.11	<0.001	0.02	0.03	0.01	0.21	<0.001	0.20	0.85	0.31	2.34	0.758
**HIV Stage (WHO)**															
Low (1&2)	1.00	1.00				1.00	1.00				1.00	1.00			
Advanced (3&4)	9.48	4.62	0.80	26.70	0.087	2.25	0.25	0.05	1.17	0.078	7.27	6.39	2.52	16.16	<0.001
**Food security**															
Secured	1.00	1.00				1.00	1.00				1.00	1.00			
Insecure	0.57	0.24	0.01	5.91	0.382	1.26	0.26	0.03	2.60	0.250	4.65	7.19	2.18	23.74	0.001
Hunger	21.87	8.64	1.26	59.23	0.028	4.40	0.29	0.06	1.55	0.139	41.03	31.71	10.28	97.86	<0.001
**Wealth Index**															
Low	1.00	1.00				1.00	1.00				1.00	1.00			
Middle	0.13	0.08	0.01	0.58	0.013	0.08	0.13	0.02	0.87	0.036	0.36	1.16	0.41	3.34	0.780
High	0.10	0.36	0.04	3.09	0.352	0.15	0.47	0.08	2.74	0.403	0.24	1.03	0.33	3.28	0.956
**Dietary diversity score**															
< 6 score	1.00	1.00				1.00	1.00				1.00	1.00			
≥ 6 score	0.20	0.21	0.04	1.14	0.071	0.23	0.57	0.15	2.21	0.416	0.39	1.11	0.45	2.74	0.822
**ART duration**															
<6 months	1.00	1.00				1.00	1.00				1.00	1.00			
6-12 months	1.31	2.36	0.34	16.51	0.386	0.21	3.82	0.69	21.12	0.124	1.40	1.78	0.47	6.71	0.398
>12 months	0.52	0.36	0.06	2.09	0.252	0.73	3.71	0.75	18.30	0.107	0.68	0.95	0.31	2.92	0.928

Adjusted for: Age, Sex, Birth weight, Diarrhea, Care givers education, Feeding frequency, HIV stage, Food security, House hold wealth index, Dietary diversity score and ART duration

Children who had diarrhea in the past six months were also more likely to be wasted (AOR = 22.49, 95% CI 3.38-149.61, *P = 0.001*). Wasting risk was generally lower among the children fed more frequently (AOR = 0.03, 95% CI 0.01-0.21, *p < 0.001*). Additionally, households with middle wealth index scores were associated with lower risk of child wasting (AOR = 0.13, 95% CI 0.02-0.87, *P = 0.036*) than were households with low wealth index scores.

Among ART-treated HIV-positive children, risk factors associated with stunting in multivariate analyses were as follows: household food insecurity (AOR = 7.19, 95% CI 2.18-23.74, *P = 0.001*); hunger (AOR = 31.71, 95% CI 10.28-97.86, *P=<0.001*); and advanced HIV stage (AOR = 5.71, 95% CI 2.32-14.05, *p < 0.001*).

## Discussion

This study revealed higher rates of underweight, wasting, and stunting among ART-treated HIV-positive children relative to HIV-negative children in Dar es Salaam, Tanzania. HIV-positive serostatus remained an independent risk factor for underweight and wasting in the adjusted analyses. Although the association between HIV status and stunting was not statistically significant in the adjusted analysis, a higher proportion of HIV-positive children were stunted than were those in the HIV-negative control group. Stunting, a chronic nutrition problem, typically results from more persistent factors, such as famine, chronic illnesses, lack of parental education, and poverty. Such factors are also persistent in the general population in Tanzania. On the other hand, wasting and underweight status stem from acute causes such as malaria, diarrhea, or opportunistic infections, which are relatively common among HIV-positive children. We could not find comparable results regarding the nutritional status of ART-treated HIV-positive children elsewhere; however, studies conducted among ART-naïve HIV-positive and HIV-negative children in Sub-Saharan Africa found HIV-positive children to have poor nutritional outcomes compared to their HIV-negative counterparts [5-7].

The differences in characteristics of ART-treated HIV-positive and negative children can account for the higher risk of underweight and wasting among ART-treated HIV-positive children. Households of HIV-positive children under ART had lower economic status, less education, and greater proportions of unemployed caregivers. Such disadvantaged socioeconomic conditions further complicate undernutrition among children. Despite the effectiveness of ART in ameliorating disease burdens, persistent socio-economic backwardness may ultimately retard the progress. In this regard, earlier studies also reported a lower education level among HIV-positive parents [36] and the corresponding associations with child underweight [23] and wasting [16].

In this study, children of households under conditions of hunger were more prone to underweight status. Hunger is a peak point in food insecurity where, even children who would normally be protected during food insecurity become victims of food shortage in hunger settings [26]. We also found a higher stunting risk among children of food-insecure and hunger-afflicted households. Previous studies have also found pronounced food insecurity among HIV/AIDS-affected households [17,37]. Both food insecurity and hunger represent a protracted lack of access to food in the needed quality and quantity [38], which limits linear growth in children. Other studies have shown similar associations of food insecurity with underweight [39] and stunting [16].

High feeding frequency was associated with lower risk of both underweight and wasting among ART-treated HIV-positive children. Inadequate caloric intake may cause catabolism through high glucagon hormone release, escalating acute weight loss [40]. The compounding effect of HIV/AIDS may further be attributed to high energy loss and poor nutrients absorption due to opportunistic infections [41], which implies a higher energy requirement for HIV-positive than HIV-negative children [42]. Other studies have reported similar results in this regard [16,18].

Diarrhea episodes during the six months prior to data collection were significantly associated with wasting in our study. Diarrhea is the commonest opportunistic condition among HIV-positive children and causes acute loss of weight through water and electrolytes, subjecting children to poor growth conditions. Another study also found an association of diarrhea with underweight and wasting among HIV-positive children [8], although the association was not statistically significant in another longitudinal study [43], and a previously conducted study in Dar es Salaam among ART-naïve, HIV-positive children had similar outcomes [7].

As expected, our study found an association between low weight at birth and an increased risk of underweight status. Other studies conducted in Sub-Saharan Africa have also found an association between low birth weight and underweight status later in childhood [23,44].

Poverty remained an underlying risk factor for underweight and wasting. Households with low economic status are less likely to access adequate food, health care, and quality education, and are also prone to preventable illnesses like diarrhea which result from an unhygienic environment; all of these factors serve to increase the risk of undernutrition. This study found a lower risk of underweight and wasting among children of middle compared to lower economic status households, as also supported by other studies [23,45].

HIV/AIDS was not associated with increased risk of stunting in the present study. This may be because of a high stunting prevalence even among HIV-negative children as reported by other population-based surveys [21,22]. Stunting represents a chronic growth retardation which stems from persistent health threats intertwined with poverty, frequent episodes of communicable diseases, low education levels, and illiteracy, all of which are persistent in Tanzania. Furthermore, stunting was also associated with advanced HIV-stage. At such stages, a child may succumb to poor linear growth even with adequate food availability due to frequent opportunistic infections [14].

These findings should be interpreted in the context of several study limitations. First, we could not ascertain serostatus for the control group by laboratory-based methods; instead, we screened out those who had tested positive or whose parent(s) had the disease or died of HIV/AIDS. Even in the unlikely event of control group contamination, we found lower rates of undernutrition than among the verified HIV-positive children under ART. Second, the cross-sectional design limits conclusions regarding the direction of causal relationships, though a case-control study on undernutrition determinants in high HIV/AIDS prevalence settings conducted in South Africa generated comparable findings [16]. Third, some measure of recall bias may have been introduced, though the use of nurse counselors was designed to ensure confidentiality and trust through provision of a comfortable interview environment.

Fourth, the significant difference in mean age between the two selected groups may limit the strength of our conclusion. While we were stringent on the under-five-years age limit, participants were randomly selected. The majority of attendees in RCHs are typically infants for immunization and growth monitoring, with older children attending less frequently; this results in a low proportion of older children. When unmatched, such wide mean differences may influence results. However, in our analyses we controlled for this important confounder by including age as a variable in both multivariate analyses.

Finally, our results cannot be generalized beyond the urban setting in which this study was based. Although a better design from a methodological standpoint may have been to include ART-naïve HIV-positive children, it is not ethically acceptable to have untreated children where specific treatment is available. Therefore, the comparative group consisted of the HIV-negative children alone. Overall, the selection of children on ART to control for potential effects on nutrition status and the inclusion of an HIV-negative control group were strengths of this study.

In conclusion, HIV/AIDS is more likely to be associated with an increased burden of child underweight and wasting even under ART in Dar es Salaam, Tanzania. Factors associated with underweight among ART-treated HIV-positive children include low birth weight, lower feeding frequency, household hunger, and low household socio-economic position. Similarly, wasting was associated with diarrhea, lower feeding frequency, and low household socio-economic position. Finally, stunting was associated with advanced HIV clinical stage, food insecurity, and household hunger. These results were obtained after controlling for several risk factors including ART duration. In addition to ongoing efforts to increase coverage of antiretroviral treatment toward prolonging HIV-positive children's lives, interventions to ameliorate poor nutrition status may be important. Such interventions should aim at promoting adequate feeding frequency [18], preventing and treating diarrhea [7,16], the use of supplementary feeding, and early and adequate treatment of opportunistic infections [6]. We also encourage the use of additional interventions, tailored to suit the specific population, beyond the application of antiretroviral therapy.

## Competing interests

The authors declare that they have no competing interests.

## Authors' contributions

BFS conceived the research questions, designed the study, conducted the fieldwork, analyzed data, and prepared the manuscript draft. KCP was involved in research proposal preparation, planning and analysis of data, and revision of the manuscript for publication. KO was involved in research proposal preparation and revision of the manuscript draft. JY was involved in research proposal development, data analysis, and manuscript revisions. LBM was involved in data collection, data base preparation, and manuscript revision. DPU was involved in research proposal development, logistic preparations for fieldwork, and data collection supervision. NPM was involved in data collection and revision of the manuscript. MJ was involved in revisions of the research proposal, data analysis, and revision of the manuscript. All authors read and approved the final manuscript draft.

## Pre-publication history

The pre-publication history for this paper can be accessed here:

http://www.biomedcentral.com/1471-2458/11/869/prepub
